# Exercise social support, barriers, and instructions in venous thromboembolism survivors: a descriptive study

**DOI:** 10.1016/j.rpth.2023.102147

**Published:** 2023-07-15

**Authors:** Julie A. Partridge, Philip M. Anton, Juliane P. Wallace, Leslie L. Lake

**Affiliations:** 1 Southern Illinois University Carbondale, Carbondale, Illinois, USA; 2 National Blood Clot Alliance, Philadelphia, Pennsylvania, USA

**Keywords:** barriers, exercise, social support, survivors, VTE, venous thromboembolism

## Abstract

**Background:**

Diagnosis of venous thromboembolism (VTE) can be a significant life event that leads to changes in physical activity and exercise. Currently, little is known about the psychosocial experiences of survivors including perceived sources of social support, exercise barriers, and instructions for exercise from medical providers.

**Objectives:**

This study aimed to explore psychosocial characteristics associated with VTE survivors’ postdiagnosis exercise. Specifically, 1) what are the main sources of social support utilized by VTE survivors for exercise, 2) what are the most significant exercise barriers (eg, physical, social, and psychological) faced by VTE survivors, and 3) what specific information relative to exercise is provided by medical professionals following diagnosis?

**Methods:**

VTE survivors (*n* = 472) were recruited through social media groups to participate in open-ended questions about psychosocial characteristics pertaining to postdiagnosis exercise.

**Results:**

VTE survivors reported multiple forms of exercise social support, although almost 1 in 4 participants reported having no support for exercise. Several postdiagnosis exercise barriers were noted, and the data indicated a wide variety of information from their medical providers regarding engaging in exercise following their diagnosis, suggesting that the unique benefits and drawbacks to these instructions should be examined in more detail.

**Conclusion:**

Although VTE survivors identified numerous categories of social support, there also exist numerous barriers, including a lack of standardized instructions for exercise. Further exploration of these characteristics is needed to better serve this population to encourage postdiagnosis exercise.

## Essentials

• Little is known about psychosocial factors associated with exercise for venous thromboembolism survivors.
• Questions addressed exercise social support, barriers, and information from medical staff.
• Most participants reported having social support; however, almost 1 in 4 reported having none.
• Almost 1 in 4 participants reported being told nothing about exercise by a medical provider.

## 1 Introduction

There are an estimated 10 million cases of venous thromboembolism (VTE) diagnosed annually worldwide and VTE accounts for the third highest number of cardiovascular deaths each year, trailing only heart attacks and strokes [1,2]. Approximately 100,000 Americans die of VTE annually—more than breast cancer, AIDS, and motor vehicle accidents combined, while another 800,000 survivors are diagnosed with VTE each year [3]. VTE survivors must learn to navigate common postdiagnosis treatments such as managing prescribed anticoagulant medications and integrating healthy lifestyle changes (eg, consistent physical activity) into their lives. Managing patient outcomes and preventing reoccurrence is of paramount importance for both patients and practitioners to mitigate the tremendous physical, social, and financial impact of VTE diagnoses.

One of the most prescribed treatment and recovery practices following VTE diagnosis is exercise (eg, walking) [4], which can be beneficial for recovery while also serving as a preventative measure against future VTE [5,6]. Research has found that exercising following a VTE event does not increase signs or symptoms of blood clots [7] and may lead to decreased risk of postthrombotic syndrome [8,9], further supporting the potential benefits of physical activity. However, there currently exists almost no formalized or systematically assessed guidelines to help prepare individuals to transition into (or return to) physical activity post-VTE diagnosis such as those found in cardiac or pulmonary rehabilitation [5]. This can lead to inconsistent or confusing instructions for patients with VTE [8], which can then result in lowered exercise adherence following VTE. Furthermore, much of the research that has assessed exercise outcomes following VTE has primarily examined physical markers (eg, VO_2_ output, shuttle walking tests, circulation, and thrombus progression), as well as general or VTE-specific quality of life measures. Currently, there is limited understanding of the impact of psychosocial factors such as psychological readiness, postclotting anxiety, anticoagulant/bleeding concerns, or social support [8] as either support or barriers on exercise motivation and adherence post VTE diagnosis, which represents a significant gap in the literature. Furthermore, previous research [10] has found that patients with pulmonary embolism (PE) lacked advice from health professionals following diagnosis. Currently, there is a lack of research on the overall psychosocial impact of VTE on exercise considerations (eg, motivation, barriers, adherence, and social support) that survivors must navigate to help prevent subsequent VTE. Suggesting exercise following a significant health-related event with no discussion of the psychosocial variables associated with that event may lead to lowered motivation and adherence [11]. This gap has the potential to serve as a significant barrier to the recovery of VTE survivors, thus underscoring the need for systematic research and application of knowledge on these topics to improve both practitioner recommendations and patient outcomes.

An important component of initiation and adoption of physical activity is social support. Support can take on numerous forms, including companionship (eg, someone to exercise with), emotional (eg, expressions of caring), and informational (eg, source of knowledge about how or when to engage in exercise). Exercise behaviors have been found to be significantly impacted by these different types of social support in children [12], adolescents [13], college and university students [14], and individuals in other chronic disease states, including type 2 diabetes [15] and cardiovascular disease [16]. Moreover, a recent study from Bastas et al. [17] found that adolescent athletes who are diagnosed with deep vein thrombosis (DVT) have a significant need for a variety of support services, indicating the potential importance of this construct for VTE survivors in a return to activity.

This study is a descriptive exploration of psychosocial variables associated with physical activity in VTE survivors. Specifically, the research questions were as follows: 1) what are the main sources of social support utilized by VTE survivors for exercise, 2) what are the most significant exercise barriers (eg, physical, social, and psychological) faced by VTE survivors, and 3) what specific information relative to exercise is provided by medical professionals following diagnosis?

## 2 Methods

### 2.1 Participants and procedure

The current research project utilized a cross-sectional, qualitative approach. The sample consisted of a total of 472 participants recruited from social media–based VTE survivor groups such as Team Stop The Clot (ie, purposive snowball sampling). Purposive sampling in qualitative research makes a deliberate attempt to seek participants with specific characteristics (eg, VTE survivors), while also allowing them to share the link for the study with others who meet the criteria (eg, snowball sampling) to increase the sample [18]. Page administrators granted permission for an online survey link (10.13039/100006947SurveyMonkey) and a brief explanation of the study to be posted. Institutional Review Board approval from the academic institution of the first 3 authors and informed consent from participants were obtained prior to data collection. Participants completed a demographic questionnaire and 3 open-ended questions pertaining to exercise following VTE diagnosis. Instructions indicated that participants could stop at any time and/or choose to decline to answer any part of the questionnaires, and thus, the numbers of respondents to each question are different and are each reported below.

### 2.2 Instrumentation

Participants completed a demographic questionnaire (age, years since initial diagnosis, self-identified sex, race/ethnicity, and type of blood clot). In order to identify current activity levels, the stages of change questionnaire [18] was utilized. It is a 5-item questionnaire used to categorize participants into stages of exercise participation (ie, “regular exercise” defined as any planned physical activity [eg, brisk walking, aerobics, jogging, bicycling, swimming, and rowing] performed to increase physical fitness performed 3 to 5 times per week for 20 to 60 minutes per session) based on the transtheoretical model [19].

Three open-ended questions addressed exercise following a VTE diagnosis. 1) Since your initial blood clot diagnosis, who has provided you with support for exercise (eg, people who encourage you to exercise, exercise with you, watch your kids so you can participate, give you information about exercising, etc.)? List as many as you want; 2) since originally being diagnosed with a blood clot, what have been your biggest barriers to exercise? (list as many as you want); and 3) what were you told about exercise by your medical provider(s) after your initial blood clot diagnosis?

### 2.3 Data analysis

Descriptive statistics were calculated for each of the demographic questions. A summative content analysis guided by grounded theory procedures [20,21] was utilized wherein data were categorized by question and authors independently read the responses. The first author coded the initial data to ensure consistency in the coding process once the second and third authors began reading the responses. Codes were developed through inductive (ie, derived from the data) analysis. Codes were then collapsed into higher order themes (ie, categories) where there was thematic overlap (eg, parents, spouses, significant others, and children were all collapsed into a theme of “family”). Consensus validation of the categories and supporting quotations continued until authors reached agreement on the themes represented by the raw data.

## 3 Results

Descriptive statistics are reported in Table 1. Overall, the sample was primarily White (*n* = 394; 90.6%) and female (*n* = 381; 87.8%). The largest percentage of respondents were in the 45 to 54 age group (*n* = 146; 33.6%). A total of 278 participants in the sample (63.8%) had experienced a DVT, while 310 (71.1%) had been diagnosed with a PE and 41 (8.7%) had also been diagnosed with other types of clots (eg, cerebral venous sinus thrombosis). The largest group of these diagnoses had occurred within the previous 1 to 5 years (*n* =141; 32.3%). Overall, 61.2% of the sample self-reported that they are engaging in regular exercise according to the stages of change questionnaire (ie, in either action/maintenance stage). Due to the qualitative nature of this study, we are presenting the overall findings from the entire sample for these questions. However, we do also present some information below for how barriers and social support categories were reported by exercisers and nonexercisers, with the caveat that these differences should be explored in greater depth in future quantitative research.

**Table 1:** Sample characteristics (*n* = 472).

Demographic classification	Categories	*N*	Percent of total sample
Gender	Women Men	41458	87.8%12.2%
Current age, y (*n* = 435)	18-24	9	2.1%
	25-34	57	13.1%
	35-44	100	23.0%
	45-54	146	33.6%
	55-64	73	16.8%
	65-74	43	9.9%
	Over 75	6	1.4%
	Prefer not to answer	1	0.01%
Type of VTE (*n* = 436)	DVT	278	63%
	PE	310	71.1%
	Other	51	11.7%
Race	White	394	90.6%
	Black/African American	21	4.8%
	Hispanic/Latino	16	3.7%
	Asian/Asian American	10	2.3%
	American Indian	6	1.38%
	Hawaiian native	2	0.5%
	Prefer not to answer	2	0.5%
	Prefer to self-identify	4	0.9%
Time since diagnosis (*n* = 436)	<6 mo	139	31.9%
	6-12 mo	66	15.1%
	1-5 y	141	32.3%
	5-10 y	37	8.5%
	>10 y	53	12.2%
Stage of change	Precontemplation	16	3.9%
	Contemplation	61	14.9%
	Preparation	82	20.0%
	Action	67	16.3%
	Maintenance	184	44.9%

DVT, deep vein thrombosis; PE, pulmonary embolism; VTE, venous thromboembolism.

Six social support categories were identified (participants could list multiple sources, and thus, the totaled percentages are >100%). The categories were as follows: family (56.2%), friends (23.8%), no one/none (23.4%), medical professionals (18.1%), training partners/groups/coaches (16.3%), and blood clot-specific support groups (in person or online, 9.8%; see Table 2). Participants who reported that they are currently regularly exercising did report a higher percentage of social support from family (64.1%), friends (32.5%), and training partners/groups/coaches (20.8%) than the overall sample, and nonexercisers reported higher levels of no support (30.8%) and lower levels from training partners (7.3%).

**Table 2:** Perceived sources of exercise social support.

Theme	Frequency	Percentage^a^	Description	Example quotes
Family	217	56.2%	Any member of traditional family structure such as spouse/partner, kids, and dogs	“I exercise with my son and my sisters” (P13) “My spouse helps me to prioritize exercise” (P50) “Son encourages via virtual communication” (P64) “My husband hikes with me twice a week” (P166) “My dad! We have been on our exercise journey together to better our health.” (P115) “My 2 dogs are my daily walk partners” (P379)
Friends	92	23.8%	Includes any friends not specifically mentioned as being part of an exercise class/group	“Friends walking with me” (P148) “I have a friend that I walk with most days” (P136) “A few close friends offered support and info” (P335)
None/no one	90	23.4%	No external social support identified; only reliant upon themselves or no one else	“I’ve had to do it myself…no one has helped me at all” (P257) “I have zero social support for exercise” (P3) “Actually, no one” (P76) “no one…I think that’s part of my problem” (P95)
Medical professionals	70	18.1%	Any member of the medical/allied health community (eg, doctor, nurse, PT/OT, and exercise physiologist)	“Dr encourages me” (P329) “Doctor, nurse, physiotherapist” (P62) “My physical therapist, my orthopedic surgeon, and my hematologist” (P119)
Training partners/running groups/coaches	63	16.3%	Individuals who are part of an exercise class/group (eg, training partners, Peloton/Strava groups)	“I have the Peloton community” (P362) “My tai chi and yoga teachers” (P90) “Pilates instructor” (P66)
Blood clot-specific support groups/Internet groups	38	9.8%	Members of any type of support groups (typically on social media platforms) dedicated specifically to VTE survivors (eg, Stop the Clot)	“Facebook support group” (P158) “Facebook pages are inspirational” (P244) “The main source of support I found was from an online support group” (P316)

*N* = 387 respondents.

Total responses = 570.

Average number of sources of support identified/participant = 1.5.

Participant numbers are indicated after each quote.

a Because participants could list multiple barriers, total percentages do not add up to 100%.

Six exercise barrier categories emerged from the data (participants could list multiple barriers, and thus, the totaled percentages are >100%). The barrier categories were physical (87.6%), emotional (25.6%), anticoagulant concerns (11.7%), social (8.2%), no barriers (7.7%), and other barriers (5.9%) (see Table 3). Participants who reported that they are currently exercising reported lower percentages of physical barriers (67.2%), and nonexercisers reported lower percentages of anticoagulant concerns (5.7%).

**Table 3:** Perceived barriers to physical activity.

Theme	Frequency	Percentage^a^	Description	Example quotes
Physical	373	87.6%	Barriers that are seen as being related to physical or physiological factors such as pain/physical symptoms, deconditioning/fatigue/stamina, or comorbidities	“Shortness of breath, fatigue, and chest pain/pressure” (P19) “Chest pain, dizziness, fatigue” (P35) “Chest pains, feeling like I’m going to collapse, severe breathing difficulties, high blood pressure…all symptoms are triggered by or worsened by exertion” (P149) “Swelling and pain in leg” (P202) “Chest pain, shortness of breath still” (P179)
Emotional	109	25.6%	Barriers based in emotional responses such as feelings of anxiety/depression, body image, self-presentation, shame/ embarrassment, and low self-confidence	“Fear of exercising with IVC filter” (P11) “I’m afraid. I don’t fully understand the origin of my clot and don’t want to have a stroke or to die” (P81) “Anxiety about letting my heart rate get too high” (P178) “PTSD and anxiety have made things more difficult” (P318) “Embarrassed at my lack of fitness” (P396) “I was so afraid to do anything. I didn’t trust my body…every little sore muscle gave me anxiety that I had another clot. Mentally it just messed with my confidence” (P181)
Anticoagulant concerns	50	11.7%	Barriers specific to use of anticoagulant medication (eg, fear of falls, bruises/bleeding, fear of response to anticoagulants)	“Risk of injury while on blood thinners” (P183) “Worry about excessive bleeding” (P124) “Caution with regard to risk of falls due to anticoagulants” (P259) “Fear of being alone on the bike. Fear of crashing while on blood thinners…” (P52)
Social	35	8.2%	Barriers based on a lack of social support (eg, informational, instrumental, emotional, companionship, and validation)	“Finding childcare” (P47) “Lack of access to gym/being able to work out with others due to fear of COVID” (P47) “No coaching from pulmonologist or cardiologist regarding appropriate maximum heart rate to avoid heart strain postclot, even when I asked repeatedly” (P386)
None/no barriers	33	7.7%	No perceived specific barriers to physical activity/exercise	“Very fortunate, I was able to continue immediately” (P381) “No barriers” (P311) “None” (P182)
Other	25	5.9%	Other barriers to physical activity including lack of time, environmental considerations (eg, weather), and low motivation	“Time, as I’m a mom of 2 and work full time” (P65) “Lack of motivation” (P67)

*N* = 424 respondents.

Total unique responses = 625.

Average number of barriers identified/participant = 1.5.

Participant numbers are indicated after each quote.

IVC, inferior vena cava.

a Because participants could list multiple barriers, total percentages do not add up to 100%.

Six categories also emerged regarding exercise advice from medical providers: positive, but nonspecific advice (28.9%); nothing/no advice given (23.5%); interoceptive-focused (15.5%); cautioned against or contraindicated (13.8%); positive, specific instructions (13%); and confusing or conflicting information (5.3%; see Table 4).

**Table 4:** Information on exercise/physical activity from medical providers postdiagnosis.

Theme	Frequency	Percentage^a^	Description	Example quotes
Positive, nonspecific	115	28.9%	Told exercise/PA is important/good, but no specific instructions for how to perform it was provided (eg, go slow, it is important to do, exercise is good for clots, and take it easy)	“I can proceed as normal” (P23) “Just that I could resume exercising” (P51) “To ‘just resume your regular activities’. Very vague to me and I’ve been trying to understand that statement” (P71) “It was important to build back up stamina and keep improving” (P372)
No advice	94	23.5%	Given no information on initiating/returning to exercise/PA	“Nothing – I was told to rest and take the medication” (P22) “Nothing. I wish I knew more of what and how much is allowed” (P235) “Nothing, unfortunately” (P239) “Not much at all” (P82) “I was told absolutely nothing. Doctors don’t know anything about blood clots except give you an anticoagulation drug and hope for the best” (P94) “I don’t remember them saying anything about exercise!” (P143)
Interoceptive-focused	62	15.5%	Instruction was centered on interoceptive feelings inherent to exercise/PA (eg, listen to your body, do what is comfortable)	“To exercise, but not overdo. It was the worst worded advice I ever received. I asked how much is too much [and] was told don’t overdo. If I could speak to that doctor again, I would give him a dressing down” (P15) “Don’t go mad and listen to your body and do what it lets you” (P57) “Listen to your body” (P125) “Listen to your body, which is terrifying” (P171) “Mainly, listen to your body which is hard because it’s a difficult scale of measure when you are used to pushing yourself” (P327)
Cautioned or contraindicated	55	13.8%	Told that exercise should be avoided or that it may have negative consequences (eg, do not exercise, do not go crazy or get hurt, avoid anything too strenuous; anticoagulant warnings)	“Don’t go crazy with any new exercise. I cannot find a knowledgeable doctor regarding DVT so I self-advocate” (P5) “Don’t fall down” (P48) “Slow down” (P75) “Don’t move more than necessary at first” (P360) “That I would never run a marathon” (P374) “Initially I was placed under restrictions with then a gradual return over 6-8 months” (P371)
Positive, specific	52	13%	Told that exercise/PA is important and good and given specific instructions or referrals for programming (eg, referral to pulmonary rehabilitation, given specific time restrictions)	“Not to do cardio for a month after diagnosis” (P139) “He gave me a program for the first few months…working up to 30 minutes at a time, 3 to 5 times a week” (P168) “No lifting more than 10 pounds, no inclines/hills” (P312)
Confusing or conflicting information	21	5.3%	Given information that conflicted among providers or was unclear (eg, different specialists gave conflicting information; lack of clarification even with follow-up questions)	“One said I can go back to it after 2 weeks, one said do what you can tolerate” (P86) “Hospital discharge instructions were pathetic! Was sent back to work after a couple days and [a] month later, all docs agreed it was too soon and limited my hours for next 3+ months. We need specific info” (P256) “One person told me I should never run again and only do light aerobic exercise. Another told me I could run as much as I wanted.” (P260)

*N* = 400 respondents.

Total responses = 400.

Participant numbers are indicated after each quote.

PA, physical activity.

a Because participants could list multiple barriers, total percentages do not add up to 100%.

### 3.1 Social support

Over 75% of this total sample identified social support mechanisms for exercise and identified an average of 1.5 sources per respondent. The largest category of social support was family, which included any member of a nuclear family structure such as a spouse/partner, children (adult or minors), or even dogs. An example of this category was, “my spouse helps me to prioritize exercise”.

Friends emerged from the data as a unique category of social support. Many respondents noted that their friends engaged in physical activity with them. This category represented individuals with an emotional and personal connection to the survivor and was considered unique from individuals who may have been providing support as part of exercise/training settings (eg, Peloton, Strava, and personal trainers). This category was conceptually separate from the friend category due to the fact that the relationship to these individuals was defined through their membership in these exercise settings (eg, “Pilates instructor”), rather than a unique interpersonal connection.

Blood clot-specific support groups were categorized as individuals who were not considered close, personal friends, or part of an exercise group, but rather, they were primarily identified as part of groups providing general support for patients with VTE following diagnosis, primarily on social media (eg, “10.13039/100005801Facebook support group”). Finally, medical providers did emerge as a category of support and included any member of the medical community (eg, doctors, nurses, and physical therapists). More specific information about this support group is discussed further below.

Although the majority of participants did identify sources of exercise social support, almost 1 in 4 participants indicated that they received no social support for exercise following their VTE diagnosis. These individuals frequently noted that this lack of support had led to difficulties in initiating or maintaining exercise (eg, “I’ve had to do it myself…no one has helped me at all” and “no one…I think that’s part of my problem”), suggesting that this lack of support may contribute to lowered exercise motivation and/or adherence.

### 3.2 Perceived barriers

Participants identified an average of 1.5 exercise barriers. A large percentage of the sample noted the negative impact of physical/physiological factors such as pain, shortness of breath (SOB), or swelling on their exercise behaviors. For example, participants noted, “chest pain, dizziness, fatigue”; “swelling and pain in leg”; and “chest pains, feeling like I’m going to collapse, severe breathing difficulties, high blood pressure…all symptoms are triggered by or worsened by exertion” as barriers to exercise participation.

Another quarter of the sample noted the presence of emotional barriers to exercise, which were centered around feelings of intense anxiety, depression, body image issues, and self-presentational concerns. Some of this anxiety was centered around what the physical symptoms noted above might be signifying. As one participant noted, “I was so afraid to do anything…every little sore muscle gave me anxiety that I had another clot. Mentally it just messed with my confidence”. In these instances, the anxiety was centered on what the physical sensations represented, rather than a physical sensation preventing exercise participation (eg, too much pain to continue), and thus, they were considered to be conceptually distinct.

Concern over the use of anticoagulant medication while exercising was also identified as a unique barrier category, with participants reporting exercise concerns over the possibility of bleeding events, as well as apprehension for exercising alone while taking anticoagulants (ie, “fear of being alone on the bike, fear of crashing while on blood thinners”) as a barrier.

Social barriers included a lack of social support such as lacking childcare, access to a workout facility, or appropriate exercise information (eg, “no coaching from pulmonologist or cardiologist regarding appropriate maximum heart rate to avoid heart strain postclot, even when I asked repeatedly”).

The final category of barriers identified by this sample was collapsed into one termed as “other” barriers and included various considerations such as a lack of time or motivation, but it represented only 5.9% of the responses.

### 3.3 Advice from medical providers

There were 2 categories in which participants reported being encouraged to exercise with the benefits touted; however, there was a distinction between being given general positive information about exercise and then also being provided specific information about exercise frequency, intensity, duration, or type. The positive, nonspecific category was identified as a discussion of the general beneficial aspects of engaging in exercise postdiagnosis but did not include specific information on how to safely navigate that space (eg, “to just ‘resume your regular activities’ [which was] very vague to me”). This was in contrast with the positive-specific category in which participants reported being given information about exercise that was both positive (eg, exercise is good for you) and specific (eg, specific information about how to safely participate; eg, “[My doctor] gave me a program for the first few months…working up to 30 minutes at a time, 3-5 times a week”). Participants were not prompted in this study to identify what type of medical provider gave them information; however, 37 individuals did indicate a specific provider (“doctor” = 21, cardiologist = 7, hematologist = 6, primary care provider = 4, nurse = 3, pulmonologist = 2, and emergency room doctor = 2).

The interoceptive-focused category included information that instructed the patient to associate with their bodily sensations following diagnosis and using that as a guideline for engaging in, continuing, or discontinuing exercise behaviors (eg, “…listen to your body and do what it lets you”). Many participants noted that this advice was generally unhelpful for them. Greater understanding of how these cues are received and interpreted is needed for a better understanding of the patient experience.

Nearly 1 in 4 participants reported that they were given no information about physical activity from medical professionals following their diagnosis, leaving them feeling uncertain of how to, or even if they should, proceed. Respondents noted, “I was told absolutely nothing” and “[I was told] nothing. I wish I knew more of what and how much is allowed”.

The confusing/conflicting information category included situations in which participants perceived that there was inconsistency in the exercise instructions they were given, either across time with 1 medical professional or among different providers (eg, conflicting information from different doctors); eg, “One person told me I should never run again and only do light aerobic exercise. Another told me I could run as much as I wanted”.

## 4 Discussion

VTE diagnosis is a significant physical, social, and emotional event for many survivors. Although activity is generally encouraged post VTE, there is currently limited understanding of how psychosocial variables may be associated with this experience. Social support, perceived barriers, and interpretations of medical advice may impact survivors’ exercise behaviors, and greater understanding of these variables is necessary to be able to develop better programming, support, and messaging for increased exercise. Overall, participants in the study reported having numerous social support mechanisms, while also experiencing barriers to physical activity, and a wide variety of physical activity messaging from medical providers following their VTE diagnosis.

A majority of VTE survivors reported experiencing social support following their diagnosis with family members, making up the largest category of support providers, particularly within the self-identified exercisers in the sample. This is consistent with existing research that family members (eg, spouses/partners, parents, and children) are often primary providers of social support for exercise [22]. Dog ownership has been found to increase the likelihood of exercise in general [23], and not owning a dog has been linked to increased risk of diabetes, hypertension, hypercholesterolemia, and depression compared to individuals who regularly walk dogs [24]. Moreover, these familial relationships have also been found to be particularly important for individuals exercising with chronic health conditions, with spousal support having been found to be of particular importance for cardiac rehabilitation patients [25] and individuals with type 2 diabetes [26].

Individuals outside of the immediate family structure were also identified as important sources of support and were represented by many types of relationships (ie, friends, workout partners, medical professionals, and support groups). Friendships have been found to play a significant role in initiating and maintaining exercise behaviors in non-VTE samples [27,28], and our results indicating that self-reported exercisers reported higher levels of friendship support suggest this as an important resource from which survivors may potentially draw assistance. Thus, it may be beneficial for VTE survivors engaging in exercise to draw on and/or develop these relationships as friends typically provide important types of emotional and companionship support that has been found to encourage exercise adherence in chronic disease conditions such as cancer [29].

Group exercise opportunities have also been found to have a significant impact on positive exercise behaviors such as adherence and positive emotion [30,31]. As more exercise groups have transitioned to online formats (eg, for convenience or as a result of COVID-19 restrictions), more challenges for group interaction have emerged, but have also been found to benefit participants [32], suggesting that exercise groups, particularly for individuals recovering from VTE, may draw important support from these sources, especially if they were previously exercising within that structure (eg, Peloton). The findings from this study support the importance of online groups (both exercise groups and traditional support groups) as a source of support that could be utilized more effectively as a mechanism for behavioral change.

The finding that almost 1 in 4 participants did not feel supported to exercise is notable. While a lack of social support certainly does not preclude one from engaging in exercise, it has been found to be a significant barrier to consistent participation and adherence, particularly in individuals with chronic disease [33,34], as well as older adults [35], and our results did suggest that nonexercisers did report higher levels of no support. If there is no social support available or perceived to be available to VTE survivors, this population may be more at risk for low levels of physical activity/sedentary behavior that could negatively impact the recovery process.

Findings from this study suggest that VTE survivors do experience a variety of exercise barriers. Physical symptoms (eg, pain, swelling, and SOB) were noted by almost 9 out of 10 participants and are consistent with exercise barriers found in previous research in which physical symptoms of patients with PE have been found to impact both participation in and modifications of exercise behaviors [36]. It is important to note that the percentage was lower in self-reported exercisers, suggesting that the specific nature of this relationship should be explored further in future research.

Emotional barriers, including post-VTE anxiety, depression, and body image concerns, were also noted by this sample. These findings are consistent with existing literature, which has noted significant emotional outcomes following diagnosis, including an avoidance of physical activity following PE due to fear [10,[37], [38], [39]], including fear of experiencing sensations that may mimic sensations that occur with VTE. This sensitivity has been found to be a significant factor in avoidant behaviors, particularly for activities that might produce similar physiological responses such as aerobic exercise [40,41]. Patients with cardiovascular disease (CVD) have been found to experience heightened awareness of interoceptive feedback, which can then lead to physical activity avoidance or a lack of behavioral persistence [42]. Rolving et al. [10] found that patients with PE struggled with bodily hypervigilance, as well as anxiety. Delineating the distinction of physical symptoms as a barrier in comparison to the fear of physical symptoms (ie, an emotional response) is important and the findings in this study suggest that further exploration of this concept in VTE survivors is needed.

Anticoagulation usage was also found to be a unique concern noted within this study and represents a specific type of anxiety tied to physical activity. Specifically, there was anxiety about exercise while on anticoagulants due to fear of bleeding events either while participating in specific activities or fear of having a bleeding event while alone, consistent with findings in a sample of adolescent athlete patients with DVT [15]. While use of anticoagulants is not necessarily a contraindication for exercise [43], currently there is little understanding of how anticoagulation medications may impact exercise behavior and these results do suggest that some survivors, particularly those who have re-engaged with exercise and thus perceive more risk, may experience concerns about their anticoagulant medication and exercise behaviors. It is important to understand more about how this barrier might be alleviated through more education on how to effectively modify activities to account for a lowered chance of bleeding, as well as through a discussion of how to feel safer while exercising alone for individuals taking anticoagulants.

It should be noted that only a small percentage of participants in this study identified time or motivation as a barrier to physical activity. It was included as a unique theme because this finding is inconsistent with existing research utilizing non-VTE populations, as time concerns or a lack of motivation are often noted as the most common barriers to physical activity [44]. This suggests that a VTE diagnosis may significantly change the prioritization of exercise behaviors and may necessitate the use of different exercise messaging (ie, a focus on how to effectively exercise, rather than techniques to “stay motivated”).

Participants in this study reported a wide variety of instructions regarding exercise from their medical providers, including almost 1 in 4 participants reporting that they were given no information pertaining to exercise following their diagnosis. This inconsistent messaging supports previous findings that PE survivors lacked advice from health professionals regarding their recovery [10]. Existing literature on physical activity counseling has supported its effectiveness in changing exercise behaviors in general settings [45]; however, the positive impact is mediated by a variety of factors, including provider confidence in giving information and perception of low patient interest in physical activity [46].

Only 13% of the sample reported being given specific forms of information about how to exercise effectively or safely following their VTE, while 5.3% indicated that they had received conflicting or confusing information. Both health care providers and patients with PE have been found to recognize the importance of a coherent approach to the rehabilitation process [5]. Structured exercise programs have been found to have increased adherence levels in comparison to self-directed exercise [47], and thus, the possibility of developing these types of programs may be beneficial to explore for patients with VTE increasing adherence and levels of physical activity, or at minimum, discussing the idea of exercise may alleviate some patient concerns and generate useful discussion moving forward.

Exercise information that focused on using physical/interoceptive sensations to dictate exercise behavior was generally indicated to be unhelpful by participants. Generally, the concept of associating or focusing on physical sensations, particularly at low-to-moderate levels of exercise, has been found to lead to positive affective responses [48]; however, this study utilized an apparently healthy population, and it remains unclear if this attention to interoceptive responses to exercise in a population of patients with VTE benefits from close attentional monitoring of body signals in the same way. Certainly, as noted above, physical cues may actually be a barrier to physical activity within a population that has experienced a significant medical event, and this messaging should be explored in more detail to better understand how this information is received and interpreted by the target audience of VTE survivors.

This study was limited to individuals who were members of VTE patient-specific social media groups, meaning that they may have unique characteristics that make them different from other VTE survivors and thus may not be representative of all survivors (eg, White and female). Although previous research has found social support to have a significant positive impact on health outcomes and treatment within self-managed health care regimens [49], the efficacy of specific types of social support may vary depending upon the needs of the exerciser and may fluctuate over time [50,51]. Given the lack of research on the benefits of specific sources of support for VTE survivors (particularly in terms of how they may be matched/needed at specific times in the recovery process), more research is needed to fully understand how these sources of support may be utilized more effectively for physical activity engagement following a VTE diagnosis. Future research should include efforts to recruit more diverse samples to increase generalizability and gain a richer understanding of the postdiagnosis exercise experience. Physical activity and exercise are important components of the VTE recovery process, both physically and psychologically. Only through understanding the patient experience can we adequately develop the messaging and support needed for increased adherence and more complete recovery.
